# Genetic predisposition to childhood obesity does not influence the risk of developing skin cancer in adulthood

**DOI:** 10.1038/s41598-024-58418-8

**Published:** 2024-04-03

**Authors:** Jay Keatley, Matthew H. Law, Mathias Seviiri, Catherine M. Olsen, Nirmala Pandeya, Jue-Sheng Ong, Stuart MacGregor, David C. Whiteman, Jean Claude Dusingize

**Affiliations:** 1 Faculty of Medicine, The University of Queensland, Brisbane, QLD Australia; 2 Departments of Population Health and Computational Biology, QIMR Berghofer Medical Research Institute, Brisbane, QLD Australia; 3 School of Biomedical Sciences, Faculty of Health, Queensland University of Technology, Brisbane, QLD Australia

**Keywords:** Basal cell carcinoma, Melanoma, Squamous cell carcinoma, Cancer epidemiology, Cancer genetics, Skin cancer

## Abstract

The relationship between body mass index (BMI) and melanoma and other skin cancers remains unclear. The objective of this study was to employ the Mendelian randomization (MR) approach to evaluate the effects of genetically predicted childhood adiposity on the risk of developing skin cancer later in life. Two-sample MR analyses were conducted using summary data from genome-wide association study (GWAS) meta-analyses of childhood BMI, melanoma, cutaneous squamous cell carcinoma (cSCC), and basal cell carcinoma (BCC). We used the inverse-variance-weighted (IVW) methods to obtain a pooled estimate across all genetic variants for childhood BMI. We performed multiple sensitivity analyses to evaluate the potential influence of various assumptions on our findings. We found no evidence that genetically predicted childhood BMI was associated with risks of developing melanoma, cSCC, or BCC in adulthood (OR, 95% CI: melanoma: 1.02 (0.93–1.13), cSCC 0.94 (0.79–1.11), BCC 0.97 (0.84–1.12)). Our findings do not support the conclusions from observational studies that childhood BMI is associated with increased risks of melanoma, cSCC, or BCC in adulthood. Intervening on childhood adiposity will not reduce the risk of common skin cancers later in life.

## Introduction

There is compelling evidence that childhood obesity is a risk factor for several cancers later in life^1^. Several observational studies have investigated possible associations with melanoma, but none have investigated the association between childhood BMI and risk of developing cutaneous squamous cell carcinoma (cSCC) or basal cell carcinoma (BCC)^2,3^.

However, assessing the potential causal link between BMI and melanoma through observational studies poses challenges, as body size can influence the risk of melanoma through multiple pathways. For example, obesity may have a direct causal effect on melanoma, with one postulated mechanism being that the increase in body surface area (BSA) as a consequence of higher body mass, leads to higher number of target cells at risk^4^. Supporting this notion, BSA has been positively associated with melanoma risk in some observational studies^3^. It is also possible that obese people receive less sun exposure than non-obese people, either because of restricted recreational physical activity time or other social factors, which may indirectly reduce their risk of melanoma.

Untangling these complex pathways through which obesity may influence melanoma risk poses challenges in observational studies, particularly when relying on self-reported measures of past sun exposure that exhibit low reproducibility. Mendelian randomization (MR) is an alternative approach that uses genetic variation in a natural experiment to investigate relationships between exposure and outcome in observational data^5^. Because genetic variants are inherited randomly and independently from other traits (given that certain assumptions are met), MR methods should, in theory, enable researchers to overcome some of the challenges encountered in observational studies. Here, we sought to use the MR approach to assess the effect of genetically predicted childhood adiposity on the risk of developing skin cancer later in life.

## Results

**Figure 1:**
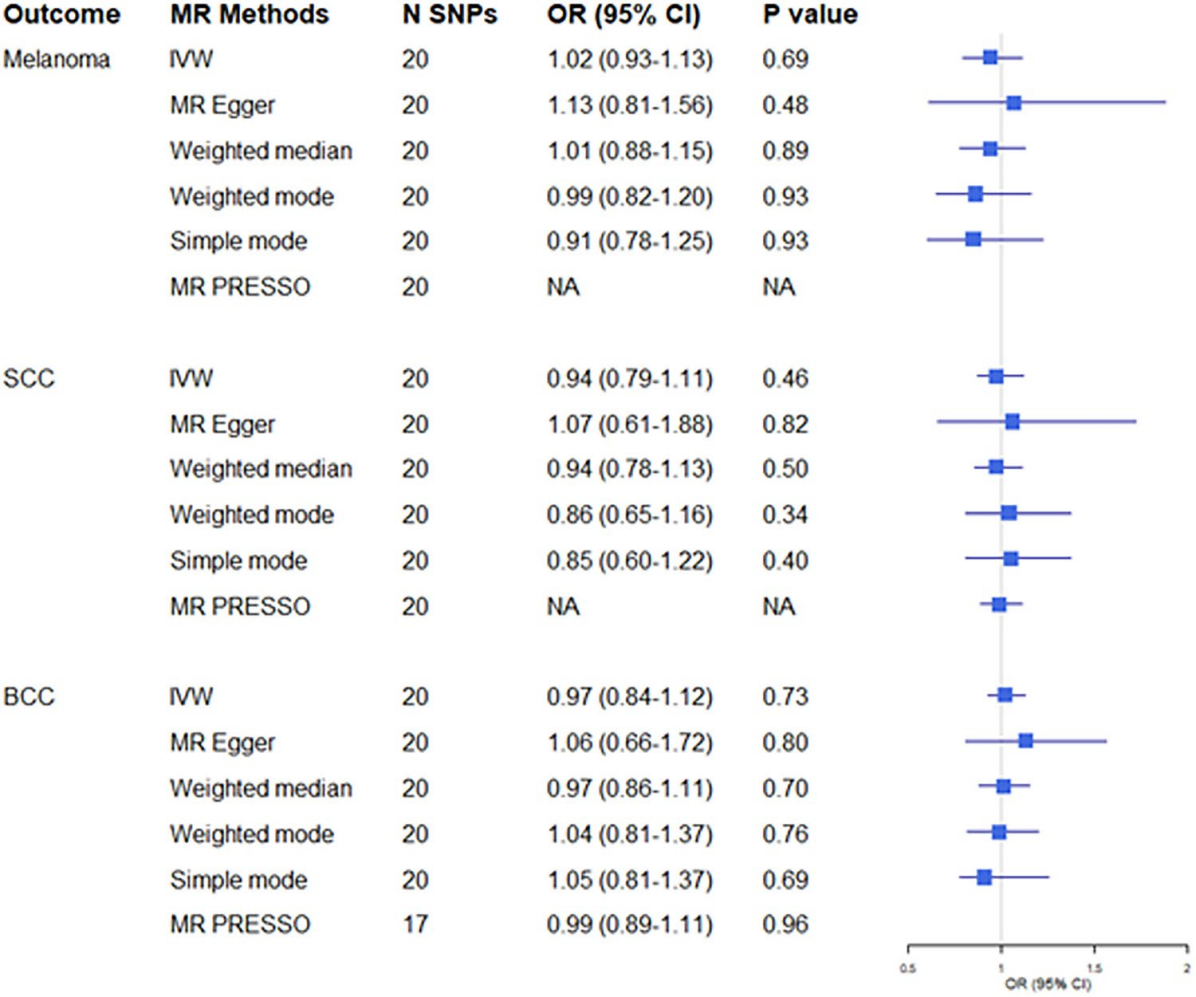
Two-sample Mendelian randomization analyses for the associations between childhood BMI and risk of developing melanoma, squamous cell carcinoma (cSCC), and basal cell carcinoma (BCC). *OR* odds ratio, *CI* confidence interval, *N SNPs* number of single nucleotide polymorphisms, *NA* The MR-PRESSO method did not detect any outlier variant for SCC and melanoma.

**Figure 2:**
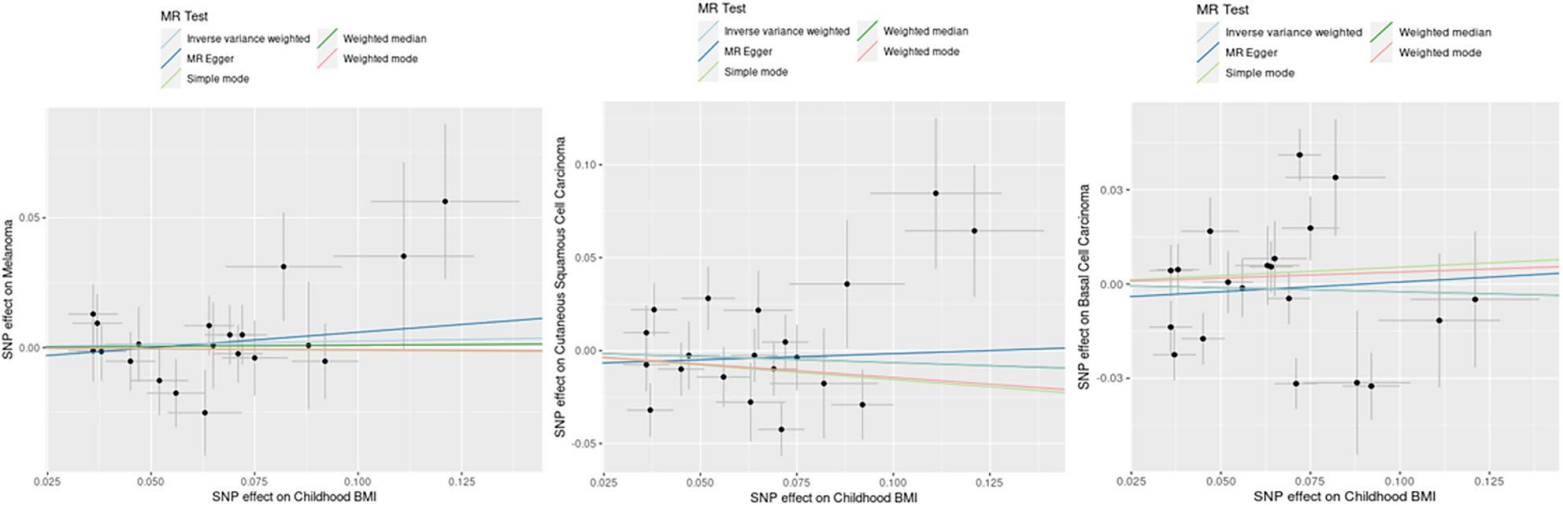
Scatter plots illustrating the causal effect of childhood body mass index (BMI) on risk of melanoma, squamous cell carcinoma (cSCC), and basal cell carcinoma (BCC). The gradients of regression lines colors correspond to the instrumental variable estimates of the effect of childhood BMI on melanoma, cSCC or BCC risk, with different MR methods compared. Error bars represent 95% confidence intervals.

**Figure 3:**
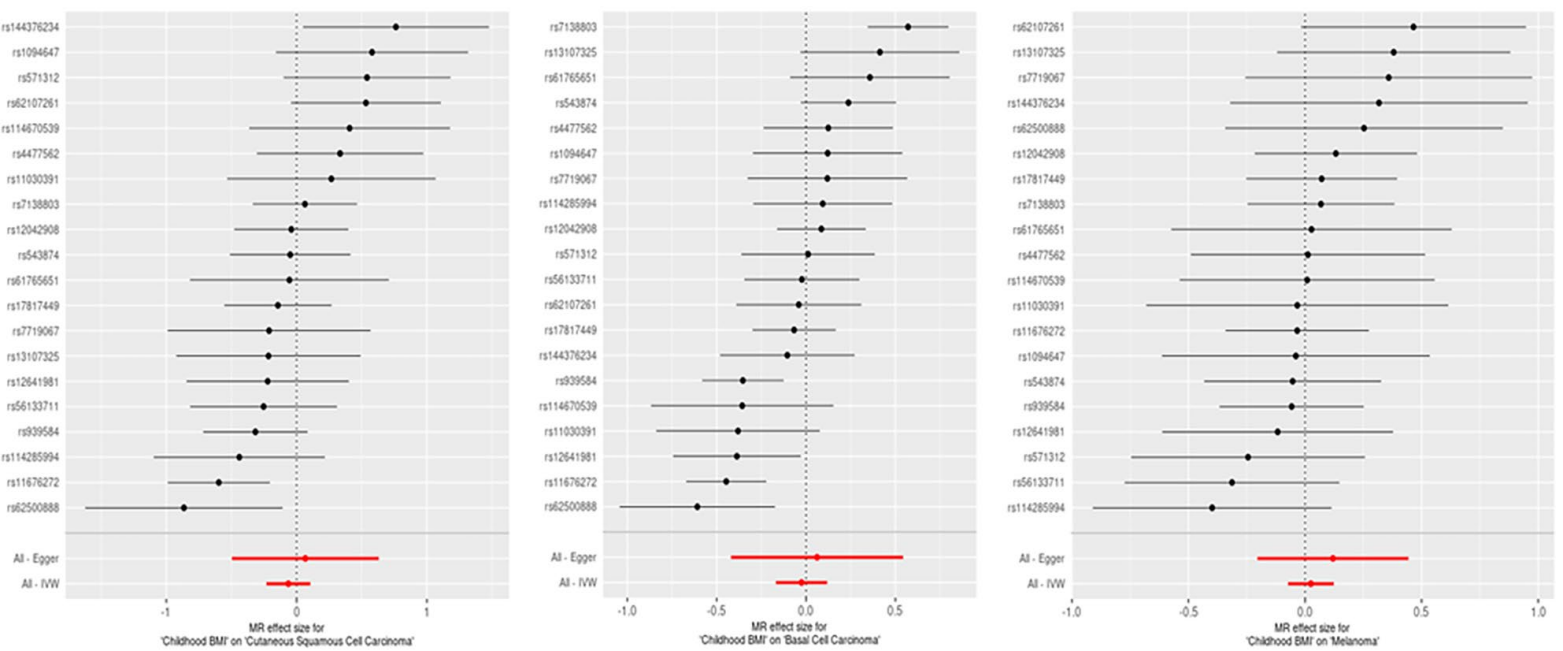
Forest plots showing the estimate for squamous cell carcinoma (cSCC), basal cell carcinoma (BCC), and melanoma using each SNP alone as well as the overall estimate using all the SNPs with MR Egger and IVW methods. The error bars represent the 95% confidence intervals.

Of the 25 genome-wide significant variants for childhood obesity, five were not available in the melanoma, cSCC, or BCC GWAS datasets, leaving 20 variants for the analyses which explained approximately 2.3% of the variance in childhood BMI. The risk estimates from the instrumental variable that combined the 20 genetic variants for childhood BMI showed no significant association between genetically predicted childhood BMI and risk of developing skin cancer. The odds ratios derived from the IVW method (OR_IVW_) and 95% confidence intervals (95% CI) per one standard deviation (SD) increase in childhood BMI were: melanoma: 1.02 (0.93–1.13), cSCC 0.94 (0.79–1.11), BCC 0.97 (0.84–1.12) (Figs. 1,2, and 3). We performed sensitivity analyses to test whether any breaches of MR assumptions may have led to the null findings; the results were consistent with estimates obtained from the MR IVW method. The MR-PRESSO method did not detect any pleiotropic variants for melanoma nor for cSCC. Three outlier variants were detected for BCC but correcting for these resulted in minimal differences to the effect estimates (Fig. 1).

## Discussion

In this MR study, we evaluated the role of genetically predicted childhood obesity on the risk of developing skin cancer in adulthood using summary data from the largest GWAS meta-analyses available. We found little evidence to suggest that genetically predicted childhood obesity has an effect on the risk of melanoma, cSCC, or BCC later in life. Indeed, the null findings provide convincing evidence that no such association exists.

There is a strong evidence that adult obesity is a risk factor for several cancers in humans^6^. The link between obesity and cancer has been hypothesized to be driven by hormonal and inflammatory factors^7,8^. Childhood obesity has also been associated with risks of developing cancer later in life^9,10^. For example, a recent MR study found an inverse association between genetically predicted childhood obesity and risk of developing breast cancer and positive associations with ovarian and colorectal cancers^11^. Thus, the hypothesis that genetically predicted childhood obesity might be linked to risks of developing skin cancer in adulthood was plausible.

While genetically predicted childhood obesity could potentially influence the risk of skin cancer through multiple pathways, our study was limited to assessing whether genetically predicted obesity might increase the risk of melanoma through a direct effect on BMI, which indirectly increases the number of melanocytes. As such, the lack of association with genetically predicted childhood obesity suggests that body size, through the mechanism of an increased BSA, is unlikely to increase the risk of melanoma and other skin cancers. Our study was unable to assess the indirect effect whereby obesity might decrease risk of melanoma by reducing outdoor activities, or other social factors. This might possibly be achieved through innovative approaches that involve using pigmentation genes as a proxy for sun exposure; however, such an approach would require strong assumptions that are not likely to be tenable.

Our MR design effectively tackles critical challenges often encountered in observational studies, specifically confounding. To enhance our ability to detect potential causal effects, we utilized genetic instruments for childhood BMI obtained from the largest genome-wide association study (GWAS) meta-analysis, which encompassed approximately 60,000 children aged 2–10 years obtained from 40 independent studies. The use of large scale GWAS summary statistics also increased our statistical power, as the two-sample MR method does not require both the exposure and outcome to have been measured in the same sample. Importantly, the childhood GWAS meta-analysis encompassed 40 studies conducted among children of European descent, allowing us to account for population stratification effectively.

Our study had some limitations. Our analyses only included participants of European descent, which limits the generalizability of our findings to other populations. It is possible that the genetic predictors of childhood obesity may differ across different populations. Whether such differences would lead to changes in risk of skin cancer remains an open question. However, given that skin cancer mostly affects people of European ancestry, this is a minor limitation. All MR analyses carry the risk of pleiotropy, whereby a genetic variant is involved in pathways that influence both the exposure and the outcome^12^. We assessed potential pleiotropy using the MR-Egger method and observed no evidence that our estimates were biased by pleiotropic variants. Lastly, while genetically predicted adiposity provides useful information, it is not a perfect proxy for directly measured childhood adiposity. This is because genetic predisposition interacts with environmental and lifestyle factors, such as diet and physical activity, which also significantly influence childhood BMI*.*

We conclude that no further investigations into the possible association between genetically predicted childhood adiposity and risk of skin cancer need to be conducted. This conclusion would only change if there was a compellingly different method for assessing adiposity in children. However, given our null findings, even if genetically predicted adiposity had an effect on risk of skin cancer, the magnitude of the effect would be very low so would likely have limited public health implications or clinical relevance.

## Materials and methods

### Summary-level genetic data on childhood BMI, melanoma, cSCC, and BCC

Genetic variants and the associated summary-level data for childhood obesity were obtained from a recent genome-wide association study (GWAS) meta-analysis^13^, which included 61,111 children (2 and 10 years) of European descent from 40 individual studies. That meta-analysis included data from multiple regions including United States, Australia, and Europe, and no significant evidence of inflation attributable to population stratification, cryptic relatedness, or other confounding factors was reported. In total, we used 25 genome-wide significant (P < 5e-8) single-nucleotide polymorphisms (SNPs) for childhood BMI in the MR analyses. We obtained GWAS summary statistics for melanoma from a GWAS meta-analysis involving 30,134 clinically confirmed cutaneous melanoma cases and 375,188 controls^14^. The BCC and cSCC GWAS summary data were obtained from GWAS meta-analyses including data from three separate datasets (UK Biobank, FinnGen, and QSkin). Briefly, fixed-effects inverse variance-weighted meta-analyses were performed using PLINK v.1.90b5.4^15^. The final analyses comprised 10,557 SCC cases and 537,850 controls and 36,479 BCC cases and 540,185 controls.

### Statistical analyses

We employed the two-sample MR approach, in which the GWAS summary data for the exposure and the outcome were selected from different (non-overlapping) samples^16^. The inverse variance-weighted (IVW) estimator was used to obtain a pooled estimate across genetic variants for childhood BMI. We performed several sensitivity analyses (MR Egger, weighted median and mode, and simple mode) to assess whether genetic variants in the childhood BMI instrument also have effects on other traits that might influence melanoma, cSCC, or BCC risk independently of the childhood BMI (i.e., pleiotropy). Scatter plot and leave-one-out analysis were used to evaluate the influence of each SNP on the overall effect estimate and to identify important outliers (data not shown). Finally, we employed MR pleiotropy residual sum and outlier test (MR-PRESSO) method to detect and correct for those outliers.

### Ethics approval

The GWAS data utilized in this study were publicly accessible and de-identified. Ethical approval and informed consent were obtained from the original GWAS, no separate ethics statement was required for this study.

## Data Availability

Genetic variants and the associated summary-level data for childhood obesity were obtained from a recent genome-wide association study (GWAS) meta-analysis by Vogelezang et al. The GWAS summary statistics for melanoma can be obtained via a direct request to the Melanoma Genetics Consortium (GenoMEL) at https://genomel.org/. GWAS summary statistics for BCC and cSCC from the FinnGen study are publicly available on the FinnGen study website (www.finngen.fi/en/access_results). Access to the raw genetic and phenotypic data used to generate the GWAS summary data for BCC and cSCC in the UK Biobank and the QSkin is possible by applying directly to the respective cohorts: UK Biobank (http://www.ukbiobank.ac.uk/wp-content/uploads/2012/09/Access-Procedures-2011-1.pdf), and QSkin (by application to QSkin Principal Investigator David Whiteman at David.Whiteman@qimrberghofer.edu.au).

## Abbreviations

BCC: Basal cell carcinoma
BMI: Body mass index
BSA: Body surface area
CI: Confidence intervals
cSCC: Cutaneous squamous cell carcinoma
GWAS: Genome-wide association study
IVW: Inverse variance-weighted
MR: Mendelian randomization
MR-PRESSO: MR pleiotropy residual sum and outlier
NHMRC: National health and medical research council
OR: Odds ratio
QIMR: QIMR Berghofer medical research institute
QLD: Queensland
SD: Standard deviation
SNP: Single-nucleotide polymorphism
UK: United Kingdom

## References

[1] Fang X (2021). Causal association of childhood obesity with cancer risk in adulthood: A Mendelian randomization study. Int. J. Cancer.

[2] Meyle KD, Gamborg M, Sorensen TIA, Baker JL (2017). Childhood body size and the risk of malignant melanoma in adulthood. Am. J. Epidemiol..

[3] Wojcik KY (2019). High birth weight, early UV exposure, and melanoma risk in children, adolescents, and young adults. Epidemiology.

[4] Nunney L (2018). Size matters: Height, cell number and a person's risk of cancer. Proc. Biol. Sci..

[5] Lawlor DA, Harbord RM, Sterne JA, Timpson N, Davey Smith G (2008). Mendelian randomization: using genes as instruments for making causal inferences in epidemiology. Stat. Med..

[6] Lauby-Secretan B (2016). Body fatness and cancer-viewpoint of the IARC Working Group. N. Engl. J. Med..

[7] Colotta F, Allavena P, Sica A, Garlanda C, Mantovani A (2009). Cancer-related inflammation, the seventh hallmark of cancer: Links to genetic instability. Carcinogenesis.

[8] Calle EE, Kaaks R (2004). Overweight, obesity and cancer: Epidemiological evidence and proposed mechanisms. Nat. Rev. Cancer.

[9] Weihe P, Spielmann J, Kielstein H, Henning-Klusmann J, Weihrauch-Blüher S (2020). Childhood obesity and cancer risk in adulthood. Curr. Obes. Rep..

[10] Célind J, Bygdell M, Martikainen J, Ohlsson C, Kindblom JM (2022). Childhood overweight and risk of obesity-related adult cancer in men. Cancer Commun. (Lond.).

[11] Gao C (2016). Mendelian randomization study of adiposity-related traits and risk of breast, ovarian, prostate, lung and colorectal cancer. Int. J. Epidemiol..

[12] Hemani G, Bowden J, Davey Smith G (2018). Evaluating the potential role of pleiotropy in Mendelian randomization studies. Hum. Mol. Genet..

[13] Vogelezang S (2020). Novel loci for childhood body mass index and shared heritability with adult cardiometabolic traits. PLoS Genet..

[14] Landi MT (2020). Genome-wide association meta-analyses combining multiple risk phenotypes provide insights into the genetic architecture of cutaneous melanoma susceptibility. Nat. Genet..

[15] Chang CC (2015). Second-generation PLINK: Rising to the challenge of larger and richer datasets. GigaScience.

[16] Burgess S, Butterworth A, Thompson SG (2013). Mendelian randomization analysis with multiple genetic variants using summarized data. Genet. Epidemiol..

